# Management of nutritional deficiencies following one anastomosis gastric bypass (OAGB): a single-center experience

**DOI:** 10.1007/s13304-025-02094-4

**Published:** 2025-01-20

**Authors:** J. Jedamzik, L. Pedarnig, C. Bichler, J. Eichelter, M. Mairinger, L. Gensthaler, L. Nixdorf, P. Richwien, N. Vock, F. B. Langer, G. Prager, D. M. Felsenreich

**Affiliations:** Division of Visceral Surgery, Department of General Surgery, Medical University of Vienna, Waehringer Guertel 18-20, 1090 Vienna, Austria

**Keywords:** One-anastomosis gastric bypass, Nutritional deficiencies, Multivitamin supplementation management, OAGB

## Abstract

**Background:**

Metabolic/bariatric surgery (MBS) remains the most effective and long-lasting treatment for obesity and its complications. Apart from any surgical complications, the often less obvious but possibly severe side-effects of nutritional deficiencies have become of interest in recent years. OAGB is known to come with the need for thorough supplementation.

**Setting:**

Retrospective study; university-hospital based.

**Aim:**

Assessing nutritional issues and their management in a real-life cohort of patients undergoing OAGB.

**Methods:**

Patients that underwent OABG between 01/2018 and 08/2019 were retrospectively assessed. Laboratory values (gained from electronic patients charts) were analyzed for nutritional issues (parathyroid hormone, vitamin A, E, D, B12, folic acid, albumin, ferritin, iron, and transferrin saturation) as well as postoperative complications and reoperations. Furthermore, weight development, improvement/remission of obesity-related complications, and regular intake of multivitamin supplementation (MVS) were assessed.

**Results:**

120 patients underwent OAGB; 89 were female. A follow-up was available for 101 patients. Mean length of follow-up was 27.8 ± 20.9 months. OAGB led to a %TWL of 36.7 ± 9.5% and %EWL of 86.8 ± 25.5%. Preoperative deficiencies were vitamin D (53.3%), followed by folic acid (16.7%) and vitamin A (6.7%). During follow-up, every patient developed at least one deficiency, hypovitaminosis D and A were predominant (74.3% and 41.0%), and 31 suffered from folic acid deficiency (30.7%). Hypovitaminosis B12 and calcium deficiency was observed in three patients (2.9%). Although advised to, only 45.5% opted for the intake of specialized MVS, whereas 10% did not take any MVS at all. More than half of all patients (53.5%) took additional supplements. Nineteen patients underwent reoperations associated with the initial OAGB.

**Conclusion:**

Two conclusions can be drawn: First, there is a general need for bypass patients to get assessed for a broad array of deficiencies over time. Second, MVS is essential for patients that had bypass surgery. Additionally, compliance needs to be promoted by educating patients as well as other treating physicians.

## Background

Obesity and its related complications, such as type 2 diabetes mellitus (T2D), arterial hypertension (AH), obstructive sleep apnea (OSA), and hyperlipidemia (HL), are increasing and affecting patients’ quality of life and life expectancy worldwide [1, 2]. While various obesity-management medications have become available in recent years, metabolic/bariatric surgery (MBS) still remains the most effective and long-lasting treatment for obesity and its associated complications [3]. Beside Sleeve Gastrectomy and Roux-en-Y gastric bypass, One Anastomosis Gastric Bypass (OAGB) is the third most common surgical procedure for patients with severe obesity [4]. The total numbers of OAGB have constantly increased over the last years to currently 7.6% of all bariatric procedures worldwide [4]. Further, OAGB is acknowledged by the International Federation for the Surgery of Obesity and Metabolic Disorders (IFSO) and the American Society for Metabolic and Bariatric Surgery (ASMBS) [5, 6].

In the literature, OAGB showed successful weight loss, remission of obesity-related complications, and good quality of life in short- and mid-term outcomes [7], nevertheless, data on nutritional deficiencies remain unclear [8]. The variability of nutritional deficiencies is affected by several factors, such as the lengths of biliopancreatic limb (BPL) and common limb (CL), pre- and postoperative patients’ education, and patients’ compliance [9, 10].

This study aims to highlight the number of different deficiencies in the blood panels of well-educated patients in a short-term follow-up and will provide evidence on the management of different types of these deficiencies.

## Patients and methods

After obtaining approval by the local ethics committee (nr. 1028/2017), a retrospective analysis of our prospectively-fed database was carried out. Patients that underwent OAGB between 01/2018 and 08/2019 were included using the electronic patients’ charts up until a follow-up visit 2 years after index surgery. All patients underwent a laboratory work-up preoperatively as well as 3 months, 6 months, 1 year, and 2 years after OAGB. Additionally, obesity-related complications at the time of operation and improvements/remissions at last follow-up were assessed for each patient as well as reoperations in the follow-up period. Furthermore, an evaluation of the following factors was carried out: sex, age, preoperative BMI [weight (kg)/height (m^2^)], % excess weight loss (%EWL) [(preoperative weight − current weight/(preoperative weight − ideal body weight) × 100] and ideal body weight (kg) [25 × height (m^2^)]; % total weight loss (%TWL) [(operative weight − follow-up weight)/operative weight × 100].

### Surgical technique

The surgical procedure of OAGB at the Medical University of Vienna is highly standardized in all patients and has previously been described in an article in a step-by-step manner [11]. Therefore, only some important key information on surgical technique will be provided at this point.

Three 12 mm trocars and two 5 mm trocars are placed under direct vision. Then, the lesser sac is opened up at the smaller curvature of the stomach about 2 cm below the incisura angularis. Next, the stomach is horizontally partially transected with a 45 mm stapler magazine. Further, the fundus of the stomach is detached from the left crus of the diaphragm. In patients diagnosed with a hiatal hernia in preoperative gastroscopy, the right and the left crura are visualized intraoperatively. If a hiatal hernia is confirmed intraoperatively, hiatoplasty is performed after complete preparation of the distal esophagus under protection of the vagal nerves. As a next step, a long and narrow pouch is created using a 36 French (12 mm) bougie introduced by the anesthesiologist. Usually, four 60 mm stapler cartridges are needed here. Then, the angle of Treitz is identified and 150 cm BPL of small bowel is measured. Further, the posterior wall of the end-to-side gastrojejunostomy is created using a linear 30 mm cartridge. The ventral defect of the gastrojejunostomy is then closed with two V-Lock absorbable sutures. Then, an “anti-reflux” suture using non-absorbable V-Lock between the BPL and the last 6 cm of the pouch is done. Finally, Petersen’s space is closed with V-Lock non-absorbable and the alimentary limb is attached to the antrum of the remnant stomach with a non-absorbable suture to prevent kinking of the alimentary limb. Finally, an air-leakage test of the gastrojejunostomy is performed with continuous air insufflation by gastroscopy before removing the trocars.

### Nutritional outcomes

For this study, the following laboratory parameters were assessed longitudinally: parathyroid hormone (PTH), vitamins (folic acid, A, E, B12, D), minerals (calcium, iron), ferritin, transferrin, transferrin saturation, hemoglobin, mean corpuscular volume (MCV), and albumin. Deficiencies were defined as plasma levels below the lower limit of normal defined by the laboratory’s cut-off levels (see Table 2) at any given point after the operation. Nutritional supplementation was assessed during each visit and was subdivided by specialized vs. non-specialized multivitamin supplementation (MVS) and additional supplementation of either Vitamin D, A, folic acid, or iron at last follow-up. All patients received the recommendation toward a specialized multivitamin formulation as a once daily dose as well as additional calcium supplementation twice daily (500 mg) within the first 6 months. Depending on the individual’s need, the calcium supplementation was continued thereafter.

### Statistical analysis

As this study includes all patients that had OAGB within the time period, a power-analysis was not necessary. Additionally, this study has a descriptive nature and does not include comparisons to other collectives.

Descriptive data in this study are presented as percentage or mean with standard deviation wherever appropriate. The comparisons of blood values before and after surgery were performed using Students *t* test with a *p* value of < 0.05 considered as significant. SPSS^®^ v28 for Windows^®^ (IBM Corporation, Armonk, New York, USA) was used for statistical calculations.

## Results

A total of 120 patients received OAGB during the observed period. Nineteen patients (15.8%) were lost to follow-up, e.g., due to missing postoperative follow-up visits. Eighty-nine patients were female, with a mean age of 46.2 ± 12.1 years and a mean preoperative BMI of 44.2 ± 4.6 kg/m^2^. Mean length of follow-up was 27.8 ± 20.9 months.

### Weight loss and remission of obesity-related complications

After OAGB, mean postoperative BMI was 28.1 ± 5.2 kg/m^2^, mean %EWL was 86.8 ± 25.5%, and TWL was 36.7 ± 9.5%. In the postoperative course, there were 22 reoperations, 19 of which were associated with primary MBS (conversion to RYGB, internal hernia, cholecystectomy). Seventeen patients suffered from type 2 diabetes at the time of operation, two of these patients experienced an improvement (only oral antidiabetic medication), and ten patients went into remission (no further medication necessary). Five patients remained on the same therapy as before OAGB (two patients on insulin therapy and three patients on oral medication). Refer to Table 1 for detailed information.

**Table 1:** Patients’ characteristics

**Sex**	Female: 89 male: 31
**Age** mean, SD	46.2 ± 12.0
< 40 (*n*)	40
> 40 (*n*)	80
**ORC**	
Arterial hypertension (*n*,%)	45 (37.5)
OSAS/COPD (*n*,%)	7 (5.8)
T2D (*n*,%)	17 (14.2)
Steatosis hepatis (*n*,%)	34 (28.3)
> 2 Comorbidities (*n*,%)	30 (25.0)
**Preoperative weight** [kg] mean (min to max)	125.7 (95; 194)
**Preoperative BMI** [kg/m^2^] mean (± SD)	44.2 (± 4.6)
**Postoperative BMI** [kg/m^2^] mean (± SD)	28.1 (± 5.2)
**TWL** [%] mean (± SD)*	36.7 (± 9.5)
**EWL** [%] mean (± SD)*	86.8 (± 25.5)
**Reoperations**	
Conversion to RYGB	11
Cholecystectomy	5
Internal hernia	3
Other non-MBS	3

*n* number of patients, *TWL* total weight loss, *EWL* excess weight loss, *SD* standard deviation, *T2D* diabetes mellitus type 2, *ORC* obesity related complications, *OSAS* obstructive sleep apnea syndrome, *COPD* chronic obstructive pulmonary disease

^*^At last time of follow-up

### Preoperative state of nutrition

Before surgery, most patients had at least one deficiency. Eleven patients had elevated parathyroid hormone, and 64 patients were suffering from hypovitaminosis D (16 of which severe, below a threshold of 30 nmol/L).

Hypovitaminosis A was found to be present in eight patients, hypovitaminosis E in three. Twenty patients had preoperative folic acid deficiency and two patients had vitamin B12 deficiency. Concerning preoperative iron metabolism, anemia (represented by hemoglobin levels below 12.0 g/dL in women and 13.5 g/dL in men) was found in nine of our patients. Low serum iron and ferritin levels were present in three patients, and 20 patients had a transferrin saturation below 12%. MCV was below normal in six patients. No patient had preoperative hypalbuminemia. Refer to Table 2 for an overview.

**Table 2:** Mean serum levels of the parameters assessed (± SD) in the postoperative time course

Parameter	Normal range	Baseline	3 m	6 m	12 m	24 m
Parathyroid hormone	15–65 pg/ml	48.6 ± 18.6 (*n* = 74)	48.6 ± 17.2 (*n* = 66)	43.0 ± 19.9 (*n* = 70)	45.3 ± 17.0 (*n* = 63)	46.1 ± 19.2 (*n* = 44)
25-OH-Vitamin D	75–250 nmol/L	49.2 ± 23.6 (*n* = 78)	73.7 ± 28.9 (*n* = 64)	78.4 ± 27.8 (*n* = 69)	67.4 ± 29.7 (*n* = 66)	65.2 ± 25.4 (*n* = 45)
Calcium	2.15–2.50 mmol/L	2.2 ± 0.1 (*n* = 74)	2.3 ± 0.1 (*n* = 63)	2.3 ± 0.1 (*n* = 62)	2.3 ± 0.1 (*n* = 63)	2.3 ± 0.2 (*n* = 44)
Vitamin B12	145–569 pmol/l	341.3 ± 147.8 (*n* = 78)	465.9 ± 228.4 (*n* = 66)	437.9 ± 207.7 (*n* = 66)	515.4 ± 257.6 (*n* = 64)	504.1 ± 262.6 (*n* = 45)
Folic acid	9.53–44.9 nmol/l	15.5 ± 10.7 (*n* = 74)	17.2 ± 10.1 (*n* = 64)	18.9 ± 12.7 (*n* = 60)	21.9 ± 14.8 (*n* = 55)	23.0 ± 14.8 (*n* = 55)
Vitamin A	1.05–2.45umol/l	1.5 ± 0.4 (*n* = 77)	1.1 ± 0.4 (*n* = 65)	1.2 ± 0.3 (*n* = 63)	1.3 ± 0.4 (*n* = 64)	1.5 ± 0.4 (*n* = 41)
Vitamin E	11.6–46.4umol/l	29.4 ± 7.6 (*n* = 75)	23.1 ± 5.5 (*n* = 66)	25.0 ± 5.8 (*n* = 64)	26.1 ± 7.1 (*n* = 64)	28.3 ± 7.5 (*n* = 42)
Albumin	35–52 g/l	42.5 ± 3.5 (*n* = 81)	42.3 ± 3.5 (*n* = 60)	41.6 ± 3.8 (*n* = 62)	42.7 ± 5.1 (*n* = 57)	42.5 ± 5.7 (*n* = 44)
Iron	33–193 µmol/l	70.5 ± 24.2 (*n* = 73)	62.3 ± 18.6 (*n* = 53)	71.8 ± 26.2 (*n* = 56)	80.5 ± 29.6 (*n* = 52)	68.6 ± 25.7 (*n* = 38)
Ferritin	men, women > 50y 30- 400 µg/L	128.4 ± 119.1 (*n* = 74)	149.5 ± 129.3 (*n* = 67)	129.0 ± 98.8 (*n* = 60)	131.8 ± 114.4 (*n* = 50)	100.5 ± 101.5 (*n* = 33)
	women 15–150 µg/L					
Transferrin	200–360 mg/dL	281.3 ± 40.2 (*n* = 74)	215.0 ± 31.1 (*n* = 67)	232.4 ± 52.0 (*n* = 60)	247.7 ± 48.6 (*n* = 50)	285.9 ± 46.7 (*n* = 33)
Transferrin saturation	16–45%	20.2 ± 8.3 (*n* = 73)	22.8 ± 11.1 (*n* = 66)	23.4 ± 8.9 (*n* = 64)	24.2 ± 11.0 (*n* = 61)	19.8 ± 9.7 (*n* = 41)
Hemoglobin	13.5–18.0 g/dL male 12.0–16.0 g/dL female	13.3 ± 2.1 (*n* = 91)	13.1 ± 1.7 (*n* = 70)	12.9 ± 1.8 (*n* = 71)	12.6 ± 1.8 (*n* = 66)	12.5 ± 1.4 (*n* = 49)
MCV	78.0–98.0fL	84.0 ± 9.6 (*n* = 91)	83.9 ± 10.6 (*n* = 70)	85.1 ± 10.9	85.5 ± 5.6 (*n* = 65)	87.4 ± 6.4 (*n* = 49)

*n* number of patients presenting with deficiency, *m* months, *MCV* mean corpuscular volume

### Postoperative state of nutrition

Within the first two years, every patient developed at least one deficiency defined by serum levels below normal. Mean serum values in this cohort were within normal range in this time period. Table 2 shows detailed data of our patients on a timeline. Twenty-one patients had elevated PTH levels, and ten patients showed persisting high levels in more than one postoperative visit. Looking at Vitamin D, deficiencies were observed in 75 patients, 56 (55.4%) of whom maintained the deficiency over time. Vitamin A deficiency was found “de novo” in 41 patients (40.6%); one patient developed hypovitaminosis E and 31 folic acid deficiency (30.7%). Thirty patients had hypervitaminosis B12 (29.7%), hypovitaminosis B12 was observed in three patients (2.9%). Low levels of ferritin were observed in 15.8% (*n* = 16) of patients, anemia was present in 35 patients (34.7%) at any time during follow-up. Fourteen patients suffered from anemia at more than one postoperative visit. Low calcium levels were found in three patients (2.9%) within the first year, only one of whom presented with calcium deficiency after the second year.

### Nutritional supplementation

Forty-six patients took a specialized multivitamin supplementation designed for patients after MBS. Twelve patients used regular MVS, ten patients did not take any MVS, and 33 patients did not provide any information regarding supplementation.

During the observed period, the majority of patients were in need of additional supplementation. Calcium is not taken into account as it is prescribed twice daily for every patient within the first 6 months and therefore not counted toward additional supplementation, and 54 (53.5%) took additional supplementation, with Vitamin D being the predominant one (50 patients). Nine patients also used additional vitamin A, 15 patients folic acid, and 17 patients iron supplements.

## Discussion

This article describes the status of deficiencies in a normal collective of patients with obesity that received OAGB and were instructed on the intake of MVS and follow-up visits in a standardized way. Pre- and postoperative vitamin levels as well as patient compliance for vitamin intake and follow-up visits were assessed. Further, this article provides readers with an overview on the current state-of-the-art treatment of different vitamin and mineral deficiencies.

### Weight loss and remission of obesity-related complications

Weight loss and remission/improvement of obesity-related complications, such as T2D, arterial hypertension, OSAS, etc., are important parameters when talking about the outcome of an MBS as well as for the comparison to other studies.

With a TWL of 36.7 ± 9.5% and EWL of 86.8 ± 25.5% after a mean of 27.8 ± 20.9 months, this cohort can be seen as very successful, having achieved exactly the amount of weight loss to be desired in this context. Thus, most patients have come in slightly above normal weight (i.e., slightly overweight but not obese) at the end of the initial weight loss process. These results are in line with the other OAGB studies reporting good weight loss [7, 12, 13]. This study has shown that a BPL of 150 cm is enough for most patients to achieve good results in the short term. Nonetheless this is a controversy as other studies found different results. The concept of tailored limb lengths was evaluated by Komaei et al. [14] comparing > 200 cm BPL vs bypassing 40% of total bowel length. This study found no difference concerning weight loss parameters with a lower amount of nutritional issues in the tailored limb length group. Yet what has to be kept in mind is that this study gives only preliminary 12 month data.

A recent meta-analysis by Salman et al. [15] including 7 studies that > 200 cm BPL has a higher weight loss as compared to 150 cm BPL at the expense of more nutritional deficiencies. Robert et al. [7] found in their randomized controlled trial a good safety profile with few nutritional complications in an OAGB with 200 cm BPL length.

In terms of remission and improvement of obesity-related complications (ORC), the results of this study are very good and comparable with the existing literature [7, 12, 13, 16]. This shows that also for this aspect, 150 cm of BPL seems to be enough for the majority of this patients. Slagter et al. found the same remission rate of ORC for limb lengths of 150 cm, 180 cm and 200 cm [17].

In summary, looking at weight loss and remission of obesity-related complications, the outcome for OAGB in this study was comparable with results from the literature favoring a 150 cm BPL length. Nonetheless, no definite answer can be given toward an optimal limb length to maximize the beneficial effects and lessen nutritional issues.

### Nutritional outcomes

One of the concerns when talking about the outcome of OAGB is the question whether nutritional deficiencies will decrease. For this reason, this study has provided pre- and postoperative levels of different vitamins, electrolytes, and other parameters. The cohort is a mixed collective of patients some of whom take their recommended MVS, some take other supplements than the recommended MVS, and some take additional MVS or no MVS at all—all of which represents a normal collective of patients after OAGB as it probably occurs in every bariatric center around the globe. This study has shown that there are slightly decreasing levels of vitamins to be found especially in the phase of the largest weight loss (3 months). After this phase, when the weight becomes more and more stable, re-increasing levels of the measured parameters can be observed. This shows that most patients seem to have adequate doses of their MVS when a stable weight is reached. Another aspect that contributes to the stabilization of blood levels might be the physiological adaption of the intestine as well as the adaption toward as higher food intake in the years after OAGB [18].

These nutritional outcomes are also comparable with the literature and lie within a normal range after MBS [16, 19]. However, it should be mentioned that studies with longer BPL generally report higher nutritional deficiency levels [9, 20].

Some studies found that tailoring the BPL as a percentage/defined part of total bowel length [14, 18] or keeping a fixed common limb [21] has favorable results concerning nutritional deficiencies than 150 cm or 200 cm BPL length.

MVS should start several weeks before MBS as deficiencies have been found in the preoperative blood panels of some patients, as mentioned earlier. Higher levels at the time of the surgery will help these patients soften the drop of vitamin levels in the early postoperative phase.

In terms on nutritional status, this study has shown that an adequate intake of the recommended MVS combined with regular follow-up visits may prevent severe problems with deficiencies.

### Patients’ compliance

Although patients are recommended to take a specialized MVS, almost half of the patients choose to either change this recommendation toward a regular MVS or even opt for no supplementation at all [22, 23]. This might also be an issue when patients that underwent MBS are followed up by a general practitioner: Vitamin and trace-element supplementation is sometimes seen as less important than medication addressing more obvious/seemingly more pressing conditions, such as arterial hypertension. This might be attributed to the more subtle or very delayed symptoms that come with the deficiency of micronutrients. Other reasons for discontinuation of the recommended MVS are gastrointestinal disturbances after MVS intake or others, such as obstipation after oral iron intake, economic reasons, or a complicated schedule when additional supplements are needed [24].

### Management of nutritional deficiencies

In case of nutritional deficiencies during follow-up, patients were supplemented according to our institutional standard. When deficiencies are detected, patients received additional supplementation addressing the missing components. To enhance compliance, a specialized MVS should be recommended to all patients [24]. Table 3 gives an overview on the institutional standard.

**Table 3:** Supplementation guidelines additionally to specialized MVS

Micronutrient	Symptoms	RDA	Supplementation OAGB	Deficiencies
Vitamin D	Bone substance loss, hypocalcaemia	600 IE Pregnancy, > 70 years of age: 800 IE	3000 IE D3/day for maintenance	3000–6000 IE D3/day 50.000 IE D2 three times/week
Vitamin A	Early: Night blindness, loss in taste Late: Blindness, keratomalacia	Men: 900 µg (3000 IE) Women: 700 µg (2300 IE)	5000–10000 IE/day	Without changes to cornea: 10.000–25.000 IE/day until clinical improvement With changes to cornea: 50.000 IE-100.000 IE/day i.m. for three days Continued with 50.000 IE/day i.m. for 2 weeks
Vitamin E	Sensoric and motoric neuropathy, ataxia, hemolytic anemia	15 mg (22.4 IE)	15 mg (22.4 IE)	90-300 mg (100–400 IE) / d
Vitamin B12	macrocytic anemia, neuropsychiatric disorders (e.g., peripheral neuropathy, depression)	2.4 µg	350–1000 µg / d orally 1000 µg / month s.c	1000 µg /day until normal blood values are achieved
Folic acid	macrocytic anemia, sensoric neuropathy, neural tube defects	400 µg	400–800 µg/d Women of childbearing age: 800–1000 µg/d;	1000 µg/d p.o. until normal blood values are achieved
Iron	Anemia	Men and postmenopausal women: 8 mg/d Women: 18 mg/d	Men, postmenopausal women, no history of anemia: 18 mg/ d as MVS Menstruating women, women/men with history of anemia 45-60 mg / d as elementary iron (any source)	150–300 mg orally 2–3/day; consider i.v. if no response
Calcium	Secondary hyperparathyroidism, bone diseases	1000–1200 mg	1200–1500 mg / day in two dosages	2000 mg /d until normal blood values are achieved

*RDA* recommended daily allowance, *i.m.* intramuscular, *d* day, *s.c.* subcutaneaously, *MVS* multivitamin supplementation

The most commonly seen deficiency is vitamin D. Vitamin D plays a crucial role in bone metabolism in an interaction with PTH and calcium blood values [25]. Even though additional supplementation is implemented, more than half of the patients persisted with low vitamin-D levels within the first two years. This might partially be attributed to low compliance. Nevertheless, a high dose of vitamin-D supplementation in addition to the MVS should be implemented to sustain normal blood values [26]. Calcium deficiency was found in 2.9% of the patients which is lower than found in a recent meta-analysis by Cao et al. [27] with deficiencies occurring in up to 7.4%. This difference might be attributed to a very strict recommendation toward calcium supplementation twice daily within the first six months at our center. Depending on serum levels and urine calcium excretion, this supplementation is continued beyond the first year after surgery.

Management of iron deficiency can be difficult due to the gastrointestinal side-effects caused by oral iron intake [28]. In this analysis, 16.8% needed additional iron, which is consistent with the literature on OAGB [29]. A general recommendation should be to avoid foods that inhibit absorption (e.g., coffee, red wine, and egg protein) when taking MVS. A possibility to further enhance intake would be using fortified food (e.g., iron enhanced salt) [30]. Oral supplementation recommended after OAGB depends on the patients’ history (e.g., preoperative anemia), age, and sex. Women of childbearing age should be recommended 45–60 mg of elementary iron. In case of adherence problems due to side-effects or inefficiency, an i.v. supplementation should be implemented. When anemia occurs, treatment becomes more complex as also vitamin B12, folic acid, vitamin A, and certain trace elements play an important role in the formation of red blood cells [31].

This study found that Vitamin B12 deficiencies, when supplemented, are rarely a problem. In case of specialized MVS hypervitaminoses B12 was very common in almost a third of the patients observed. All this might be attributed to the long-lasting depots in the body as studies with patients undergoing gastrectomy have found that Vitamin B12 levels deteriorate as late as 20 months [32].

Depending on the institutional standard of laboratory testing, some deficiencies will only be detected by clinical symptoms. For example, vitamin B1 is not common in the laboratory screening in our facility, but can cause severe neurocognitive symptoms up to Wernicke’s encephalopathy [33]. The treating physician needs to be aware of these deficiencies as immediate supplementation with vitamin B1 is warranted to prevent any worse outcome.

Not mentioned in this analysis yet also important for any treating physician are the individuals’ life choices such as specific eating habits (e.g., vegetarians/vegans) as well as age, sex, level of physical activity and hormonal status, all of which may influence the supply of micronutrients.

### Limitations of the study

As this article is based on a retrospective analysis, it comes with all limitations typical for this type of study. With 16% (19 patients) being lost to follow-up within the first two years, these could not be included in the analysis, but might be of genuine interest in detecting deficiencies. Furthermore, our laboratory panel does not include Vitamin B1, selenic acid, zinc, and copper, which can also cause severe clinical problems. An important limitation is also that patients’ compliance in our study was analyzed retrospectively. We cannot be sure whether patients actually do take the additional supplementation prescribed when deficiencies occur. Additionally, we did not address any sex differences in the supplementation and hormonal status during blood testing. Nonetheless, this might also play an important role in interpreting some findings.

## Conclusion

To sum up, two conclusions stood out in this study: First, there is a general need for the assessment of deficiencies by a broad laboratory panel. Second, specialized MVS should be prescribed to all patients undergoing bypass surgery. Both need to be done more thoroughly within the first two years after the bypass procedure, as this is the phase of highest weight loss when severe deficiencies are most likely to occur. Micronutrient supplementation can be difficult in patients with low compliance. Hence, continuous education for patients but also other professionals working in this field needs to be provided.

## Data Availability

The authors confirm that the data supporting the findings of this study are available within the article.
